# Comparison of off-clamp microwave scissors-based sutureless partial nephrectomy versus on-clamp conventional partial nephrectomy in a canine model

**DOI:** 10.3389/fsurg.2023.1255929

**Published:** 2023-09-19

**Authors:** Ha Ngoc Nguyen, Atsushi Yamada, Shigeyuki Naka, Ken-Ichi Mukaisho, Tohru Tani

**Affiliations:** ^1^ Department of Advanced Medical Research and Development, Shiga University of Medical Science, Shiga, Japan; ^2^ Department of Urology, Faculty of Medicine, University of Medicine and Pharmacy at Ho Chi Minh City, Ho Chi Minh City, Vietnam; ^3^ Medical Innovation Research Center, Shiga University of Medical Science, Shiga, Japan; ^4^ Department of Surgery, Hino Memorial Hospital, Shiga, Japan; ^5^ Division of Pathology, Shiga University of Medical Science, Shiga, Japan

**Keywords:** partial nephrectomy, off-clamp, microwaves, renal function, renal ischemia

## Abstract

**Objectives:**

To compare the usefulness and safety of off-clamp microwave scissors-based sutureless partial nephrectomy (MSPN) with on-clamp conventional partial nephrectomy (cPN) in dogs.

**Methods:**

We performed off-clamp MSPN using microwave scissors (MWS) in six dogs, and on-clamp cPN in three dogs, in two-stage experiments. The bilateral kidney upper poles were resected via a midline incision under general anesthesia. After 14 days of follow-up, the lower pole resections were performed. The renal calyces exposed during renal resections were sealed and transected using MWS in off-clamp MSPN and were sutured in on-clamp cPN. In the off-clamp MSPN group, the generator's power output of MWS was set as either 50 W or 60 W for each kidney side. We compared the procedure time (PT), ischemic time (IT), blood loss (BL), and normal nephron loss (NNL) between the two techniques using the Mann–Whitney *U*-test.

**Results:**

We successfully performed 24 off-clamp MSPNs and 12 on-clamp cPNs. The off-clamp MSPN was significantly superior to on-clamp cPN in avoiding renal ischemia (median IT, 0 min vs. 8.6 min, *p* < 0.001) and reducing PT (median PT, 5.8 min vs. 11.5 min, *p* < 0.001) and NNL (median NNL, 5.3 mm vs. 6.0 mm, *p* = 0.006) with comparable BL (median BL, 20.9 ml vs. 23.2 ml, *p* = 0.804). No bleeding and major urine leakage were noted during the reoperations.

**Conclusions:**

Off-clamp MSPN outperforms on-clamp cPN in lowering the risks of postoperative renal function impairment in dogs.

## Introduction

Partial nephrectomy (PN) has become the “treatment of choice” for T1 renal cell carcinoma (1) since it achieved a similar oncologic outcome (2) to that of radical nephrectomy. Whereas, PN was superior to radical nephrectomy in preserving renal function (RF) (3) and reducing risks of cardiovascular disorders, which could contribute to the superiority of overall survival reported in large real-world databases (4).

PN conventionally involves hilar clamping and tumor removal followed by renorrhaphy (5). Hilar clamping can reduce blood loss and bring a clear surgical view that helps in accurate tumor excision. However, reducing renal ischemia and reperfusion injury demands a short clamping time and thus requires resecting the renal parenchyma quickly, repairing the collecting system if needed, and closing the parenchyma by suturing in a short time. Such fast suturing occasionally injures renal vessels, causing delayed bleeding, artery pseudoaneurysms, and arteriovenous fistula formation (6). Although hemostatic agents are used conveniently in renorrhaphy to reduce hemorrhage (7), their effects on other renovascular complications are limited. Moreover, they are foreign materials and still carry risks of infection and allergic reactions.

Sutureless PN is an alternative procedure in which the resected bed is ablated and sealed using energy devices such as radiofrequency sealers (8) and coagulators (9), ultrasound sealers (10), laser probes (11), microwave probes (12, 13), and so on, to control renal bleeding. These devices obtained effective outcomes for patients with small and superficial tumors. However, sutureless PN with a short clamping time for large and highly complex tumors is still challenging due to their suboptimal device tip shapes and insufficient sealing function.

Recently, microwave scissors (MWS), which install a microwave irradiation function into mechanical scissors (14), have enabled operators to perform tissue microwave coagulation, vessel sealing, and mechanical cutting smoothly. The MWS was designed as a scissor-shaped surgical instrument, suitable for use in both open and laparoscopic surgery. Therefore, operators can naturally employ their skills and techniques, similar to using traditional scissor instruments. Excellent clinical track records have been reported in partial pancreatectomies (15), lung segmentectomies (16), colectomies (17), and thyroidectomies (18). Microwaves intrinsically induce dielectric heat by oscillating the dipoles such as water molecules in tissues. This heating process is more direct and faster than that induced by other energy forms, making them an excellent energy source for tissue coagulation (19). These reports suggest a hypothesis that the MWS could have a sufficient sealing function in organs with high blood perfusion, such as kidneys, and the use of MWS can enable PN to be performed in short/zero ischemic time.

We propose and evaluate a novel sutureless PN technique without hilar clamping using MWS. In this study using a canine model, we evaluate the feasibility of off-clamp microwave scissors-based sutureless partial nephrectomy (MSPN), compare its usefulness and safety with on-clamp conventional partial nephrectomy (cPN), and assess the histopathological changes of renal tissue after thermal injury induced by MWS.

## Methods

### Microwave scissors

**Figure 1:**
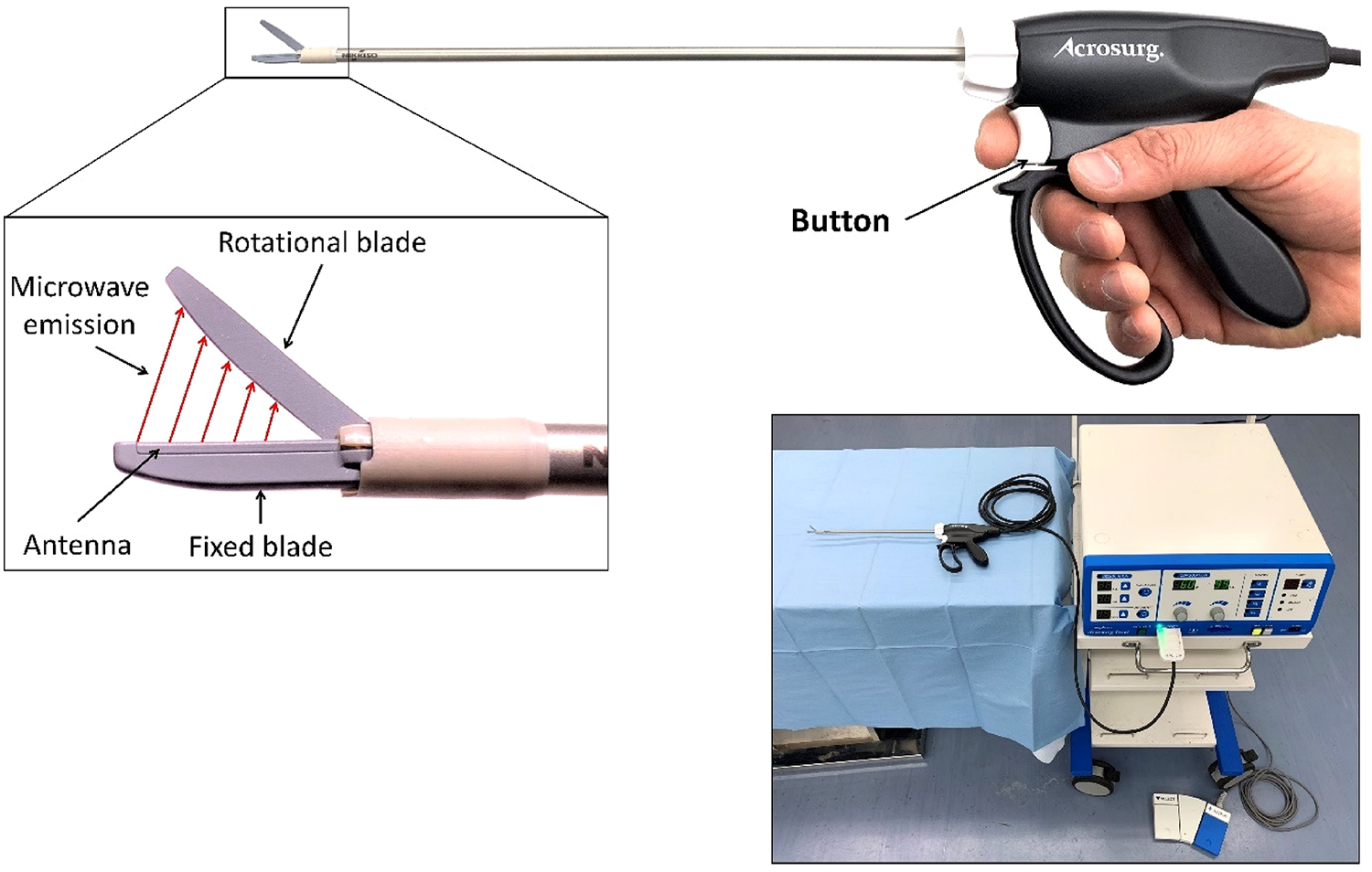
The microwave scissors and their generator (right-lower corner).

The MWS (Acrosurg Revo S, Nikkiso Co., Ltd., Tokyo, Japan) is shown in Figure 1. The fixed and rotational scissor blades are the extension of the inner and outer conductors (14) of the microwave-transmitted coaxial cable connected to a 2.45 GHz microwave generator. Microwaves are emitted from the fixed blade to the rotational blade while pushing the button or the footswitch, creating an alternating electric field on the tissue placed between the scissor blades. This electric field intrinsically induces dielectric heat by oscillating the dipoles such as water molecules at a frequency of 2.45 GHz, causing direct tissue coagulation without heat sink effects (19). The microwave irradiation time and cutting timing can be arbitrarily adjusted (14), allowing the MWS to be used flexibly and adaptively as cold scissors, scissors for cutting with seamless sealing, or a simple microwave coagulator without cutting. When the operators gently grasp tissue using scissor blades, irradiate microwaves, and then mechanically cut them, the MWS can seamlessly seal the tissue like bipolar radiofrequency and ultrasonic sealers (18, 20). When the operators close or partially open the scissor blades, and touch the scissor-blade side to the bleeding tissue while irradiating microwaves (18, 21), the interblade microwave irradiation induces an electric field around the scissors, causing dielectric heat that can coagulate tissue and stop bleeding.

### Animals and surgeries

Eleven beagles weighing approximately 10 kg each raised in a pathogen-free environment were used for this study. We used the first two dogs to assess the feasibility of off-clamp MSPN and to determine the generator's power outputs that are suitable for the kidneys. The pilot-phase data was not included in this article. In the main-phase study, nine dogs were divided into two groups: (1) the off-clamp MSPN group in which PN without hilar clamping or renorrhaphy using MWS was performed in six dogs, and (2) the control group involving three dogs for on-clamp cPN.

The experiments were performed in two stages under general anesthesia. For the first stage, each dog was placed in a supine position. The upper poles of both kidneys were resected via a 15-cm-midline incision. After 14 days of follow-up, we reoperated the dogs to inspect for postoperative complications and perform bilateral lower pole resections as the second stage. All procedures were performed at the level of either the upper or lower polar line of each kidney. At the end of the second stage, we performed euthanasia for remnant kidney sampling.

**Figure 2:**
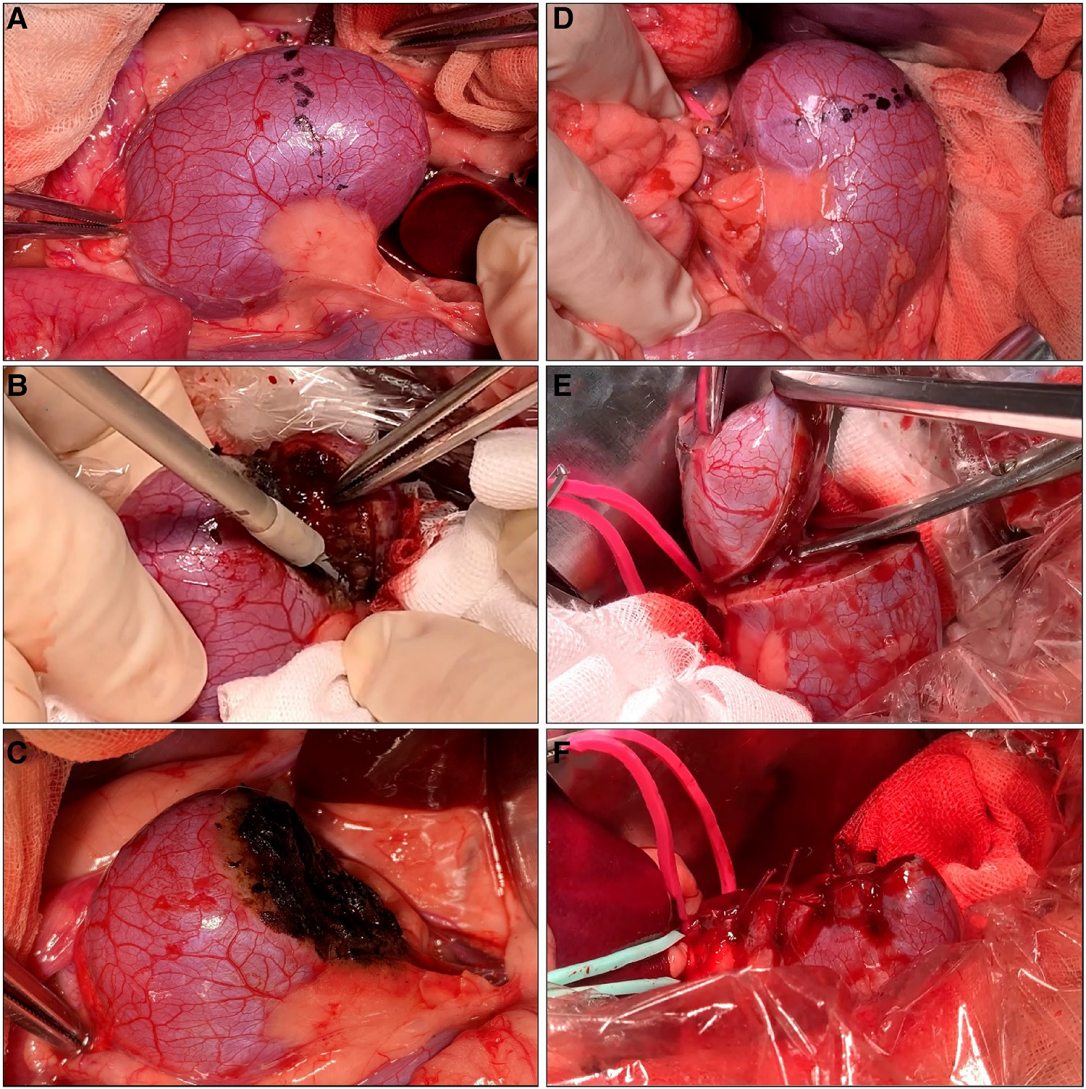
Off-clamp microwave scissors-based sutureless partial nephrectomy (MSPN) (**A–C**) and on-clamp conventional partial nephrectomy (cPN) (**D–F**) for upper pole resections. The excision line was marked at the level of the upper polar line (**A**,**D**). The kidney's upper poles were resected using microwave scissors (MWS) (**B**) without hilar clamping in off-clamp MSPN or using Metzenbaum scissors (**E**) after hilar clamping in on-clamp cPN. The resected beds were coagulated using MWS without renorrhaphy (**C**) in off-clamp MSPN, or sutured (**F**) in cPN.

For off-clamp MSPN, we performed renal resections using only MWS without hilar clamping or renorrhaphy, as shown in Figures 2A–C. The generator's power output of MWS was alternately set at either 50 W or 60 W for each kidney side. We subgrouped every three dogs to perform off-clamp MSPN with or without precoagulation. In the non-precoagulation subgroup, we used the MWS to bite and seal the renal parenchyma and then cut them mechanically while slightly lifting the resected tissue using forceps with the other hand. The renal calyces and vessels exposed during renal resections were sealed and transected using MWS. The resected bed, if oozing, was recoagulated using MWS to consolidate hemostasis. In the precoagulation subgroup, we coagulated the excision line using MWS before performing the same manner described above to minimize the BL.

For on-clamp cPN, we performed hilar clamping and renal resection followed by suturing as shown in Figures 2D–F. Initially, we separately clamped the renal artery and vein using bulldog clamps (FB330R, Aesculap B-Braun, Melsungen, Germany). The renal parenchyma was resected using Metzenbaum scissors. Finally, we performed renal suturing in two layers. We sutured the renal medulla and the opening of renal calyces using a running suture (Monodiox 3-0, Alfresa Pharma, Osaka, Japan). The renal parenchyma was reconstructed using interrupting sutures (Opepolyx-N 2-0, Alfresa Pharma, Tokyo, Japan). Additional sutures were carried out if bleeding persisted after hilar declamping.

### Outcome measurements

We recorded kidney size, kidney volume resected (KVR), ischemic time (IT), procedure time (PT), blood loss (BL), and normal nephron loss (NNL) of the remaining kidneys. The estimated kidney volume (EKV) was calculated using the ellipsoid sphere volume formula, $V=\frac{4\Pi }{3}d_{1}d_{2}d_{3};$ where *d*_1_, *d*_2_, and *d*_3_ indicate the length, width, and thickness of an ellipsoid sphere, respectively. The percentage of kidney volume resected (%KVR) was determined by dividing the KVR by the EKV. The PT was counted from the beginning of renal resection until the bleeding from the resected bed was completely controlled, using MWS in off-clamp MSPN or by renorrhaphy in on-clamp cPN, respectively. The IT was the clamping time in on-clamp cPN. The BL was determined by subtracting the preoperative weight of dry gauze from the postoperative weight of the corresponding blood-soaked gauze after each procedure. The NNL was determined as the largest depth measured from the resection line to the edge of either the thermal injury zone in the renal remnants induced by MWS-based coagulation in off-clamp MSPN or the suturing zone in on-clamp cPN, as observed in histopathological images.

The dogs were monitored for postoperative complications during 14 days of follow-up after the first-stage experiment. During the second stage, aspects of the intra-abdominal condition, such as remnant kidney status, ascites, hematoma, and internal bleeding from the upper pole resections in the first stage, if any, were recorded.

### Histopathological evaluation

To assess the macroscopic features of the lateral thermal injury induced by MWS in off-clamp MSPN and the devascularization zone induced by renorrhaphy in on-clamp cPN, the renal remnants were sectioned perpendicular to the resected bed. Hematoxylin and eosin staining was performed for histopathological evaluation.

### Statistics

Data were analyzed using SPSS Statistics for Windows, version 22.0 (IBM Corp., Armonk, NY, USA). The non-parametric Mann–Whitney *U*-test was used to confirm the differences in medians between the two quantitative groups. Statistical significance was defined by a *p* value <0.05.

## Results

### Perioperative results

We successfully performed 24 off-clamp MSPNs and 12 on-clamp cPNs. The perioperative outcomes of the two groups are presented in Table 1. All dogs survived after 14 days of follow-up. Off-clamp MSPN was significantly superior to on-clamp cPN in avoiding renal ischemia (median IT, 0 min vs. 8.6 min, *p *< 0.001), shortening PT (median PT, 5.8 min vs. 11.5 min, *p *< 0.001), and reducing NNL (median NNL, 5.3 mm vs. 6.0 mm, *p *= 0.006) with comparable BL (median BL, 20.9 ml vs. 23.2 ml, *p *= 0.804).

**Table 1:** Perioperative outcomes of off-clamp MSPN and on-clamp cPN in dogs.

Parameter	Off-clamp MSPN (*n = 24*)	On-clamp cPN (*n = 12*)	*p* value*
KVR (g), median (range)	4.5 (2.9–6.0)	4.6 (3.5–6.0)	0.402
%KVR (g), median (range)	15.2 (9.4–24.0)	16.2 (11.1–20.6)	0.908
PT (min), median (range)	5.8 (3.1–13.4)	11.5 (8.2–13.5)	<0.001
IT (min), median (range)	0 (0–0)	8.6 (7.8–10.6)	<0.001
BL (mL), median (range)	20.9 (8.0–130.4)	23.2 (13.1–45.7)	0.804
NNL (mm), median (range)	5.3 (3.8–6.6)	6.0 (5.2–8.7)	0.006
Calyceal sealing/suturing, *n* (%)	16 (66.7)	7 (58.3)	–
Urine leakage, *n*	0	0	–
Postoperative bleeding, *n*	0	0	–

KVR, kidney volume resected; %KVR, percentage of kidney volume resected; PT, procedure time; IT, ischemic time; BL, blood loss; NNL, normal nephron loss.

* Mann–Whitney *U*-test.

Except for two procedures in the non-precoagulation subgroup that had an outlier BL (68.2 ml and 130.4 ml), all other off-clamp MSPNs had BL of <45.2 ml. The renal parenchyma was torn during renorrhaphy in one on-clamp cPN, and required additional sutures, resulting in a deeper NNL (8.7 mm). The renal calyx was seamlessly sealed using MWS or sutured to prevent urine leakage in 16 off-clamp MSPNs (66.7%) and 7 on-clamp cPNs (58.3%). No complications such as bleeding and major urine leakage (recognized by ascites appearance) were noted during the reoperations.

Subgroup comparison demonstrated that precoagulation caused a significantly lesser BL compared to non-precoagulation in off-clamp MSPN (median BL, 17.5 ml vs. 33.5 ml, *p* = 0.028). The PT and BL in the 50-W subgroup were greater than those in the 60-W subgroup, albeit without statistical significance. Off-clamp MSPN resulted in a significantly shallower NNL compared to cPN, as observed on both day 0 (median NNL, 5.4 mm vs. 6.2, *p* = 0.049) and day 14 (median NNL, 5.1 mm vs. 5.9 mm, *p* = 0.049) postoperatively.

### Tissue changes after MWS-based coagulation and renorrhaphy-induced devascularization

**Figure 3:**
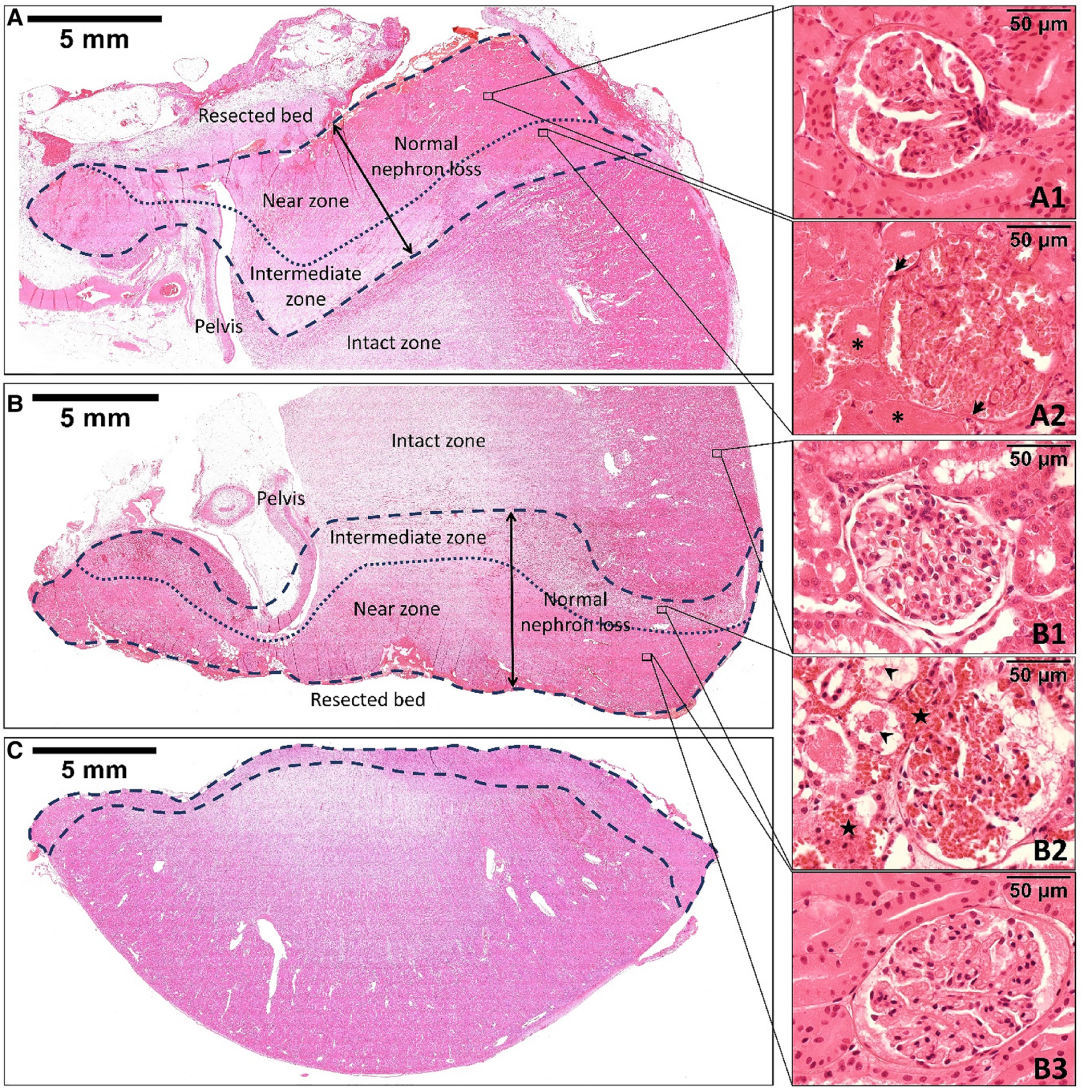
The hematoxylin and eosin staining of the remnant kidney's upper pole at the two-week follow-up (**A**), the remnant kidney's lower pole (**B**), and the resected specimen (**C**) immediately after off-clamp microwave scissors-based sutureless partial nephrectomy. The area limited by the dashed line indicates the thermal injury zone. The dotted line divides the thermal injury zone into two zones: the near zone and the intermediate zone. The morphology of the renal tissue in the near zone was well maintained for up to two weeks postoperatively (**A1**, **B3**). However, the renal tubular cells and glomeruli were slightly smaller than those in the intact zone (**B1**). The intermediate zone (**B2**) was characterized by the extravasation of erythrocytes (stars) into interstitial spaces. The renal tubular cells were sporadically ruptured and detached into the lumen (arrowheads). Two weeks postoperatively, the intermediate zone (**A2**) exhibited coagulative necrosis that was characterized by the disappearance of tubular cells’ nuclei (asterisks), infiltration of macrophages and neutrophils (arrows), and fibrosis formation.

Both microwave coagulation and renorrhaphy result in tissue necrosis. Figure 3 shows the hematoxylin and eosin staining of the resected specimen and the remaining kidney after off-clamp MSPN. The MWS-induced thermal injury included two zones: (1) the near zone, which had closer contact with the scissor blades, and (2) the intermediate zone, which separated the near zone from the intact zone. Although the morphology of renal glomeruli and tubules including nuclear staining (Figures 3A1,B3) was well maintained for up to two weeks postoperatively in the near zone, we noted that the cell membranes disappeared, the cytoplasm looked homogeneous, and erythrocytes were completely disrupted. These properties were not found in the intact zone (Figure 3B1). The intermediate zone (Figure 3B2) was characterized by the extravasation of erythrocytes into interstitial spaces, and the renal tubular cells that were sporadically ruptured and detached into the lumen. Two weeks postoperatively, the intermediate zone exhibited coagulative necrosis that was characterized by the infiltration of macrophages and neutrophils, as well as degeneration of the renal glomeruli and tubules with nuclear disappearance, and fibrosis (Figure 3A2).

**Figure 4:**
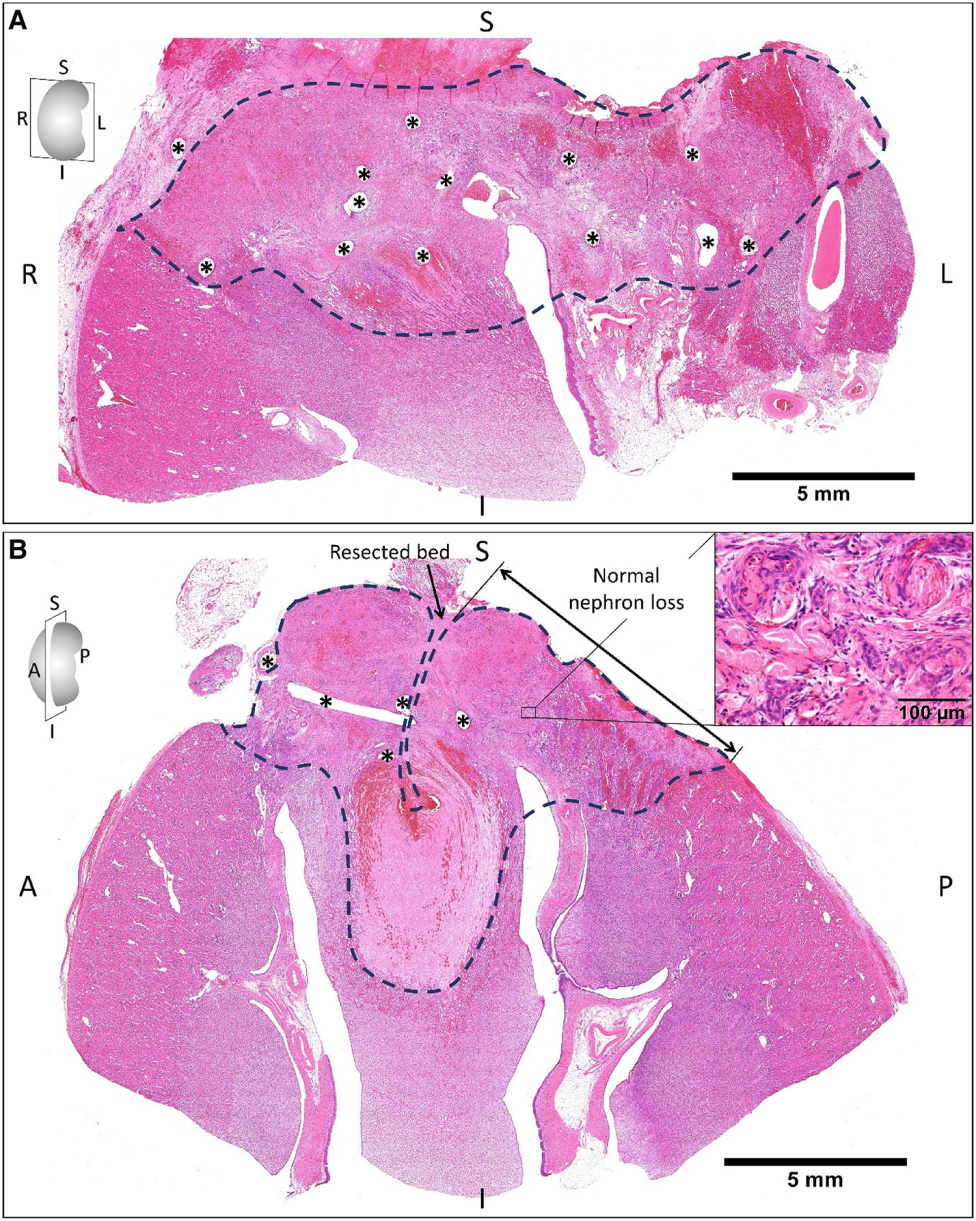
The hematoxylin and eosin staining of the remnant kidney sectioned on coronal (**A**) and sagittal planes (**B**) two weeks after on-clamp conventional partial nephrectomy. The areas limited by the dashed line indicate the devascularization zone induced by renorrhaphy (asterisks are suturing holes), which exhibited blood congestion and tissue necrosis (**B**, right-upper corner) with the infiltration of macrophages and neutrophils, degeneration of renal glomeruli and tubules, and fibrosis. S, superior; I, inferior; R, right; L, left; A, anterior; P, posterior.

On the other hand, the suturing zone observed two weeks after operations (Figure 4) exhibited blood congestion and tissue necrosis with the infiltration of macrophages and neutrophils, degeneration of renal glomeruli and tubules, and fibrosis. We noted that the nuclei of renal tubular cells were completely lost.

## Discussion

In this initial assessment in dogs, off-clamp MSPN was performed faster than on-clamp cPN with comparable BL and lesser NNL, albeit without hilar clamping. These findings demonstrated that MWS-based coagulation can adequately control renal bleeding in canine off-clamp PN without the requirement for renorrhaphy or hemostatic agents. In addition, we provided an understanding of the two-week renal tissue changes after thermal injury induced by MWS compared to the devascularization caused by renorrhaphy.

### Off-clamp sutureless PN vs. on-clamp cPN with renorrhaphy

In on-clamp cPN, the hilar clamping-induced renal injury and the healthy parenchyma loss after surgery are responsible for the RF decreases (22), and were considered modifiable targets to preserve postoperative RF. Although no differences in RF decreases were found between off-clamp and on-clamp robotic PN in patients with two kidneys, regular baseline function, and tumors with a RENAL score ≤10 (23, 24), off-clamp is superior to on-clamp PN in preventing acute kidney injury and new-onset stage ≥3b chronic kidney disease in solitary kidney patients (25, 26). These findings suggest that on-clamp PN with limited renal ischemia is acceptable in patients with two normal kidneys (24). However, hilar clamping should be avoided when technically feasible for patients with solitary kidneys or low baseline RF (25, 26).

Mir et al. (27) analyzed pre- and postoperative renal parenchymal volume using computed tomography imaging and reported that a median of 83% (interquartile range: 75–91) of functioning parenchyma was preserved after PN. A strong correlation was observed, with the percentage of parenchymal volume saved being the strongest predictor (*p* < 0.001) of the percentage of glomerular filtration rate saved. In other words, healthy parenchyma loss is primarily responsible for RF decrease after surgery. Healthy parenchyma loss involves non-neoplastic parenchyma excised with the tumor and normal volume loss induced by devascularization/ablation in the renal remnant (22). Currently, enucleation or resection of a thin rim along the plane of the tumor pseudo-capsule is sufficient to achieve a negative surgical margin (28). As a result, non-neoplastic parenchyma excised with the tumor does not significantly impact postoperative RF (29). Therefore, normal volume loss induced by devascularization/ablation may dominantly contribute to RF decrease after PN. Indeed, modifying the reconstruction technique, ideally reducing normal volume loss, significantly improves postoperative RF (30).

In our study, the calculation of normal volume loss was not technically feasible because of limited facilities. Therefore, we evaluated the parameter “NNL”—the largest depth of normal volume loss measured from the resection plane, as shown in Figures 3, 4. The present study demonstrated that coagulation of the resected bed using MWS significantly reduced NNL compared to renorrhaphy. Consequently, we consider that off-clamp MSPN can reduce the risks of RF impairment by avoiding renal ischemia and preserving healthy parenchyma. On the other hand, renorrhaphy might be hastily terminated in the race against the clamping time without adequate hemostasis of the resected bed. The renal parenchyma might be torn during renorrhaphy, requiring additional deeper suturing. Furthermore, the suture needle occasionally transects the renal vessels, resulting in renovascular complications (6). Considering these challenges, we believe that employing MWS to control renal bleeding in off-clamp MSPN could reduce the procedural burden, time consumed for suturing, and perioperative complications by eliminating the need for renorrhaphy.

### The microwave thermal effects on renal tissue

We note that the histopathological changes of renal tissues after MWS-based coagulation are similar to those found in the livers after microwave ablation therapy (31, 32) reported previously, in which the morphology of the hepatocytes near the microwave coagulator was well maintained under light microscopy. However, the electron microscopy revealed serious damage to the nuclear and cytoplasmic membranes, with no apparent organelle structures such as mitochondria or endoplasmic reticulum (32). These findings could be explained that because the microwave dielectric heating was so rapid (19), tissue temperature in the near zone quickly reaches ablation range (33, 34) of 50°C–95°C. This immediately causes protein denaturation, rupture of phospholipid membranes, and destruction of cytoplasmic organelles and enzymes, resulting in irreversible cell death, whereas the tissue's structural outline was fixed. We believe that this fixation effect has relevance to the excellent hemostasis ability of MWS on kidneys observed in the present study. The absence of enzymic digestion (32) in the near zone resulted in the well-maintenance of renal tissue morphology for up to two weeks postoperatively.

In the intermediate zone, although the thermal effect gradually attenuates based on the negative temperature gradient, tissue temperature in the hyperthermia range (33) of 40.5°C–47°C, which is majorly induced by heat conduction (34), results in changing of the cell membrane permeability, leading to overaccumulation of metabolites and intracellular fluid shifts, subsequently causing cytolysis (33). Moreover, DNA and cytoplasmic organelle dysfunction secondary to protein aggregation and unfolding induced by hyperthermia (33) leading to cell death.

### The perspective and limitations of PN using MWS

Sutureless PN, which reduces technical burden by eliminating the need for renorrhaphy, is worthy of further research and development. Although several studies (8–13, 35, 36) have reported the feasibility of off-clamp sutureless PNs, these techniques limit the targets to small and low-complexity renal tumors. Brassetti et al. (36) demonstrated that the sutureless approach significantly increases trifecta achievement (negative surgical margin, no major complications, and no significant RF deterioration) compared to renorrhaphy in off-clamp robotic PN. However, the selection bias, where most patients in the sutureless group had small and uncomplex tumors, limits the generalizability of these findings. Currently, no off-clamp sutureless PN techniques are widely accepted by most urologists. On the other hand, on-clamp cPN can adequately achieve oncologic control with limited BL, for most patients with localized renal tumors, remaining the gold standard approach in nephron-sparing surgery (5).

We investigate a novel sutureless PN utilizing only MWS for renal resection and bleeding control. In this experimental study, we compared off-clamp MSPN vs. on-clamp cPN to primarily assess the capacity of MWS for controlling renal bleeding without renorrhaphy. Although the usefulness of off-clamp MSPN was demonstrated, its procedural success might be attributed to the open-surgery modality because the MWS was omnidirectionally manipulated, facilitating tissue coagulation and bleeding control in a short time. To inspect the impact of degrees of freedom on MWS manipulation in the minimally invasive surgery modality, we conducted a preliminary experiment in five kidneys from three pigs (21) following the present study. It demonstrated that off-clamp sutureless laparoscopic partial nephrectomy (LPN) using MWS is feasible for both middle and lower pole resections, mimicking various tumor locations in clinical scenarios. All pigs survived after three days of follow-up.

Even though hilar clamping was not performed, the off-clamp sutureless LPN using MWS (21) recorded shorter PT and lesser BL compared to porcine on-clamp LPNs (37, 38), in which renal bleeding was controlled with renal suturing (37, 38), renal suturing with hemostatic agents (38), or electrocautery with hemostatic agents (37), respectively. In addition, its BL (21) was lesser than that of off-clamp open PN using ultrasonic (39) or radiofrequency ablation devices (39), in similar porcine renal resections reported previously. To our knowledge, the cost of MWS is lower than that of other devices usually employed for dissection in conventional LPN, such as bipolar radiofrequency or ultrasonic devices. Consequently, we consider that the use of MWS can provide an affordable surgical treatment to improve patients' outcomes not only in open PNs but also in LPNs. Moreover, if the MWS was installed into surgical robots, the realized “off-clamp MWS-based sutureless robotic PN” could provide dexterous and precise manipulation of MWS like MSPN in open surgery shown in this study with microwave coagulation-based excellent renal bleeding control.

It is crucial in MSPN that the surgeons sufficiently coagulate the tissue before cutting and meticulously control the renal bleeding to maintain a clear surgical view during resections because the MWS does not have a feedback mechanism to monitor tissue conditions during the coagulation process. The lack of tissue-condition monitoring might result in the possibility of cutting tissue after premature coagulation, leading to improper vessel sealing and bleeding. Furthermore, stopping massive bleeding might require most of the irradiated microwave energy, which can diminish the coagulation effects of the MWS on tissues and eventually obscure the resection line.

This study remains several limitations. First, we did not evaluate the pre- and postoperative RF. It is necessary to evaluate the RF decrease of the affected kidneys to reach a definite conclusion on the functional benefits of the novel method. Second, a pyelogram was not performed. Although no major urine leakage confirmed the postoperative calyceal sealing effects, the pyelogram may provide further information such as extravasation, and pelvic stenosis complications. Third, it is necessary to compare off-clamp MSPN with off-clamp PN without MWS to evaluate the advantages of MWS compared to other devices and bleeding control methods. Finally, dog kidneys are smaller and not as well vascularized as human kidneys, limiting the translation of these findings to clinical scenarios. Therefore, additional studies in human-size animals are warranted.

In conclusion, the present study provides fundamental knowledge of renal tissue changes after thermal injury induced by microwaves. In this assessment in dogs, off-clamp MSPN outperforms on-clamp cPN in shortening PT and lowering the risks of RF impairment. MWS-based coagulation can adequately control perioperative renal bleeding in off-clamp canine PN without the need for renorrhaphy. We believe that PN using MWS is a promising surgical treatment modality for patients with localized renal tumors.

## Data Availability

The raw data supporting the conclusions of this article will be made available by the authors, without undue reservation.
